# Proactive Initiative to Establish a Dengue‐Free Factory Assessment Framework in Dengue High‐Risk Industrial Areas: A Narrative Review

**DOI:** 10.1155/ipid/4901627

**Published:** 2026-09-06

**Authors:** Wanapa Ritthison, Adisak Bhumiratana, Chanthana Padungtod, Narongsak Tongtummachart

**Affiliations:** ^1^ Office of Disease Prevention and Control, Region 6 Chonburi, Chonburi 20000, Thailand; ^2^ Thammasat University Research Unit in One Health and EcoHealth, Thammasat University, Rangsit Campus, Pathumthani 12121, Thailand, tu.ac.th; ^3^ Faculty of Public Health, Thammasat University, Rangsit Campus, Pathumthani 12121, Thailand, tu.ac.th; ^4^ Department of Disease Control, Bureau of Occupational and Environmental Diseases, Ministry of Public Health, Nonthaburi 11000, Thailand, moph.go.th

**Keywords:** dengue, Dengue-Free Factory Assessment Framework, dengue-free workplace, industrial areas, larval source management

## Abstract

Dengue poses a growing threat in endemic countries where industrial expansion intersects with *Aedes* vector‐breeding habitats. Factories and manufacturing premises often generate a multitude of unmanaged water‐holding containers—including water storage tanks, machinery components, landscaping features, and recyclable waste—that favor perennial reservoirs for *Aedes aegypti* vectors. Despite substantial public health burdens, occupational dengue prevention in the workplace remains largely reactive, fragmented, and insufficiently aligned with safety, health, and environment (SHE) management systems. This narrative review synthesizes evidence on dengue epidemiology in industrial contexts, larval source ecology in factory environments, workers’ dengue risk perception, and the regulatory landscape governing occupational health in dengue‐endemic countries. Drawing on these evidence domains, we propose the conceptual foundations of a proactive “Dengue‐Free Factory Assessment Framework” (D‐FAF) designed to prevent and reduce *Ae. aegypti* breeding and high dengue risk in workplaces in industrial areas, safeguard employee health, minimize work loss, and achieve a sustained reduction in dengue incidence. The framework integrates the “Plan‐Do‐Check‐Act” (PDCA) logic of ISO 45001 and ISO 14001 into a scalable, standards‐aligned tool applicable across diverse industrial sectors in dengue high‐risk areas.

## 1. Introduction

Dengue is a mosquito‐borne viral infection caused by one of four genetically related dengue virus serotypes (DENV 1 to DENV 4), which is transmitted to humans through the bite of infected *Aedes* vectors, primarily *Ae. aegypti* and *Ae. albopictus*. This widespread viral disease is more common in tropical and subtropical than temperate climates [1, 2]. DENV causes a broad spectrum of clinical manifestations, ranging from asymptomatic or undifferentiated fever (estimated at 40%–80%) to symptomatic dengue fever (DF) (estimated at 20%) to severe dengue (estimated at ≤ 5% of symptomatic cases), such as dengue hemorrhagic fever (DHF) or dengue shock syndrome (DSS) [3–6]. While the majority of DENV infections are asymptomatic or DF, DENV can occasionally cause severe cases (DHF/DSS) and even death (estimated at < 1%). Infection with one of four distinct DENV serotypes provides lifelong homotypic immunity to that specific serotype but only provides heterotypic immunity with short‐term protection against others, lasting approximately 1–3 years. Factors influencing severe dengue can be increased by a subsequent DENV infection, age, and specific viral strains [7–9].

Dengue becomes significant as one of the most rapidly expanding vector‐borne diseases of the 21st century [1, 2, 5, 10]. About half of the world’s population, living in more than 130 countries, is at risk of dengue, and 100–400 million infections occur annually [5]. In recent years (2023–2026), the 2024 dengue epidemic reached an unprecedented global peak, affecting over 100 countries on all continents, particularly with over 14.1 million reported cases (a 2‐fold increase from 2023 with approximately 6.5 to 7 million cases) and over 9500 deaths (a 1.36‐fold increase from 2023 with approximately 700 deaths) [5]. Following the 2024 dengue epidemic worldwide, the ongoing trends (2025–2026) elicit the decrease of dengue case numbers in some regions (particularly in the Americas) in early 2025, but likely high transmission of dengue continues across multiple subregions in the Americas, Africa, and the Pacific. In early 2026, dengue continues to be a high‐risk, global public health threat with significant, ongoing outbreaks in nontraditional regions in Europe. Global estimates of the annual economic cost of dengue range from USD 8.9 billion to over USD 40 billion when productivity losses, hospitalization costs, and vector control expenditures are aggregated [11, 12].

The global spread of dengue is strongly associated with the global expansion of dengue vectors, likely due to their ecological plasticity [1, 4, 10, 13]. Factors that can drive the spread of DENV transmission include climate change, mosquito adaptation, travel and trade, and urbanization [1, 2, 10, 13, 14]. Climate change is strongly related to the adaptation of *Aedes* vectors due to increased temperatures, higher humidity, and rainfall patterns that allow *Aedes* vectors to thrive and expand their geographic regions, leading to the spread of dengue into higher latitudes. For example, industrialized countries in Europe like France, Italy, and Spain have recently reported locally acquired cases. Travel and trade can accelerate increased global movement that helps spread DENV to new areas, including parts of Europe [14, 15]. High‐density, unplanned urbanization can provide ideal breeding conditions for *Aedes* vectors. Such driving forces can contribute greatly to its burden that is concentrated across Asia, Latin America, and sub‐Saharan Africa.

Historically, dengue control strategies primarily focus on reduction and elimination of *Aedes* breeding habitats in urban rather than industrial environments [16, 17]. Consequently, an often‐overlooked dimension of dengue transmission is the contribution of industrial, commercial, and residential premises to the proliferation of the *Aedes* vector population [16–18]. Industrial settings such as factories, warehouses, processing plants, and construction sites frequently accumulate a diverse array of water‐holding containers for *Aedes* vectors associated with production processes, infrastructure maintenance, landscaping, and solid waste management.

Thus, in the context of industrial expansion and rapid urbanization in high‐risk dengue transmission zones, industrial workers may face dual occupational and community‐level exposure. However, the occupational health dimension of dengue prevention is rarely codified in standard safety, health, and environment (SHE) management systems. The absence of a structured workplace‐level dengue assessment tool means that even factories operating within comprehensive ISO 45001 (Occupational Health and Safety) or ISO 14001 (Environment) frameworks may lack standardized criteria for evaluating dengue risk, monitoring larval indices, or enforcing corrective actions [19–21]. This gap is particularly acute in Southeast Asia, South Asia, and Latin America, where dengue endemicity overlaps with dense concentrations of export‐oriented manufacturing industries [22].

Against this background, the present narrative review aims to (1) characterize the epidemiological and ecological dimensions of dengue risk in industrial workplace environments; (2) examine the behavioral and perceptual factors that perpetuate vector breeding in factory settings; (3) map the regulatory and standards landscape relevant to workplace dengue prevention; and (4) synthesize these evidence streams into the conceptual foundations of a proactive “Dengue‐Free Factory Assessment Framework” (D‐FAF) that can be operationalized, validated, and disseminated across dengue high‐risk industrial areas (see Supporting File A). Review methods and synthesis for this narrative review can be seen in Supporting File B. By grounding the framework in established theories of occupational health management, behavioral science, and vector control practice, we seek to provide both a conceptual road map and a practical tool for advancing dengue prevention in industrial settings, particularly confined within industrial areas such as industrial zones, industrial estates, and industrial parks (see Supporting File A).

The following questions need to be addressed here:

1. Why does dengue remain a paradoxically “persistent” public health challenge?
2. Why does dengue incidence continue to be rising despite ongoing, intensified control efforts?
3. How will uncontrolled, unplanned rapid urbanization in, or surrounding, industrial zones create perennial reservoirs for dengue?
4. How will larval source management (LSM) work in industrial environments?
5. What is the concept of D‐FAF and its operationalization?

## 2. The Paradox of Dengue Persistence

The epidemiological landscape of global dengue is characterized by a paradox. Dengue’s incidence has tripled over the last 30 years, reaching over 5 million cases in 2023 and up to over 14 million cases in 2024 [1, 5, 14, 23]. As of early 2026, global dengue cases remain high, following a record‐breaking 2024 with over 14 million reported cases. The dengue burden is heavily concentrated in tropical and subtropical regions, with Asia—particularly South Asia and Southeast Asia—accounting for approximately 70% of the total global disease burden [23].

Dengue remains a paradoxically persistent public health challenge in endemic countries due to a combination of complex viral dynamics and immunological factors [24–27]; lack of specific treatments and vaccine limitations [28, 29]; environmental and climate drivers [30–32]; vector adaptability and resistance [33, 34]; rapid, unplanned urbanization and infrastructure gaps [35, 36]; and migration and human mobility [32, 37–39] (Supporting Table 1). Although continuations of vector control efforts should logically decrease disease transmission, controversially, why do control measures fail? Meanwhile, as vector control intensifies, the population’s herd immunity may diminish, paradoxically leading to more frequent and intense outbreaks.

## 3. Dengue in Industrial Areas

### 3.1. Factors Driving Reservoirs for Dengue in Industrial Areas

**FIGURE 1:**
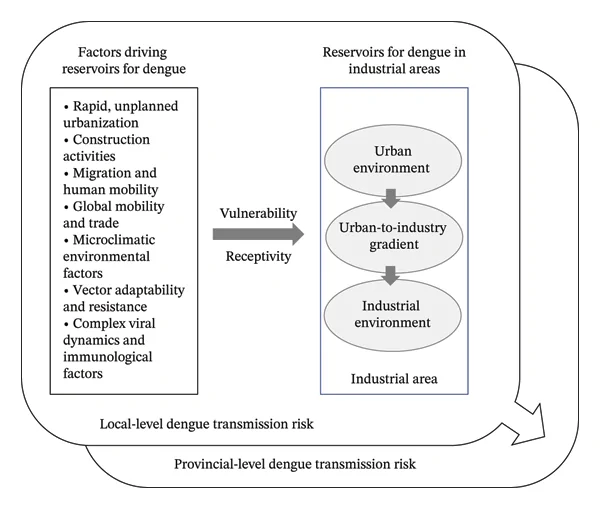
Factors driving reservoirs for dengue in industrial areas.

Unplanned urbanization in or surrounding industrial areas is a global phenomenon, particularly prevalent in developing countries facing challenges of the paradox of dengue persistence, where rapid industrial expansion outpaces urban planning, infrastructure development, and regulatory oversight. Urban or industrial areas grow faster than their infrastructure can create the resulting gaps—specifically in low‐quality housing, inadequate sanitation, poor waste disposal, and poor water storage practices (Supporting Table 1). This questions how uncontrolled, unplanned rapid urbanization in or around industrial zones/estates/parks (Supporting File A) will create perennial reservoirs for dengue. It is likely that these zones act as anthropic‐hazards, where industrial activities and inadequate infrastructure merge to sustain DENV even during nonpeak transmission months. If accompanied by key factors contributing to perennial reservoirs for dengue, this creates a perpetual breeding cycle of *Ae. aegypti* in the absence or insufficiency of vector control efforts, gradually creating local‐ to provincial‐level dengue transmission risk (Figure 1).

### 3.2. Industrial Concentration and Dengue Vulnerability in Thailand

In Thailand, dengue has remained hyperendemic since the 1950s, with epidemic cycles recurring every two to 5 years [40]. As compared to other health regions, Health Region 6—covering eight eastern provinces, including dense industrial areas with high population/worker density and extensive industrial expansion with rapid urbanization—represents the highest dengue‐burden provinces. For example, Rayong and Chonburi still face a significant “dengue‐persistent” public health challenge due to high‐endemic dengue clusters (2011–2020 data). Similar to Malaysian industrial corridors and Vietnamese export processing zones [27], regional dengue transmission intensity may be attributed to high population and workforce density, international worker populations with variable dengue immunity backgrounds, and extensive industrial expansion with rapid urbanization and improper sanitation infrastructure.

## 4. *Ae. aegypti* LSM in Industrial Settings

### 4.1. LSM Strategies

In practice, dengue vector control by making use of LSM will work in an industrial setting. It refers to the physical environment, backdrop, or surroundings dominated by industry, which could span an entire neighborhood or a workplace context (i.e., occupational conditions characterized by industry). As guided by WHO [41, 42], LSM refers to the application of larval control measures applied to or used in deliberate environmental management of breeding habitats that support the proliferation of *Ae. aegypti* vector population in industrial environments. Here, LSM encompasses four principal modalities: **source elimination** (permanent removal of potential breeding containers), **source reduction** (prevention of water retention of potential breeding containers), **biological control** (introduction of larvivorous fish), and **chemical control** (application of WHO‐approved larvicides to unavoidable water bodies).

Within the context of industrial settings, source elimination is often achievable for discretionary water‐holding receptacles such as disused equipment, packaging debris, and waste containers through enhanced solid waste management protocols and recyclable material segregation programs, as described later. However, functional water utilities—cooling towers, fire suppression systems, and drainage infrastructure—cannot be eliminated and instead require engineering modifications to prevent *Ae. aegypti* infestation or re‐infestation. These include installation of tightly fitting screens or lids on water storage tanks, regular flushing of drainage channels, installation of backflow preventers in cooling water systems, and application of autocidal ovicidal or lethal ovitraps at receptive locations [42]. Biological control using *Bacillus thuringiensis israelensis* (Bti) or *Bacillus sphaericus* (Bs) formulations represents a highly specific and environmentally compatible tool for containing *Aedes* larvae in ornamental water features, drainage sumps, and other large unavoidable habitats on factory grounds [43]. Thus, the deployment of LSM in conjunction with dengue vector surveillance tools—including standard larval indices such as the Breteau index (BI), container index (CI), and house index (HI) adapted for industrial settings, particularly confined within industrial zones/estates/parks [44]—would constitute the entomological monitoring pillar of D‐FAF as described later in detail.

### 4.2. *Ae. aegypti* Ecology and Container Typology in Industrial Settings

**FIGURE 2:**
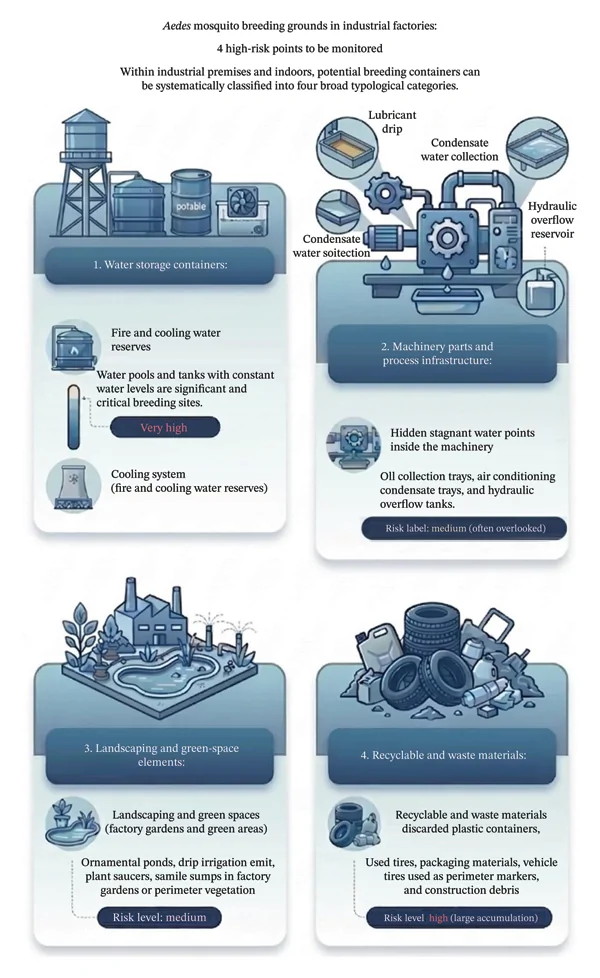
Container typology for breeding *Aedes* vectors in industrial environments.

Given its extreme ecological plasticity, *Ae. aegypti* adapted well to urban and industrial settings, exploiting year‐round water‐holding containers and human‐seeking behaviors [16, 18, 22, 45–49]. Within industrial indoors and premises, potential breeding containers can be systematically classified into four broad typological categories (Figure 2). First, **water storage containers** encompass overhead tanks, ground‐level reservoirs, fire suppression cisterns, cooling system sumps, and potable water barrels maintained for process or utility purposes. Fire suppression cisterns are engineered water storage tanks, lagoons, or underground cisterns that provide a reliable water supply for fire sprinkler systems, hydrants, and foam systems. It is an ideal place for breeding *Ae. aegypti* because the systems often require proper, consistent water level control to prevent issues like pump failure. Cooling system sumps are used to collect and manage water in industrial processes, such as cooling towers or heating, ventilation, and air conditioning systems. It is also an ideal *Ae. aegypti* breeding site because these systems generally include pits or basins, pumps, and level controllers (sump pump floats) to maintain proper water levels. These containers serve as large‐volume habitats for *Ae. aegypti* that may support high larval densities and function as persistent breeding sites irrespective of seasonal rainfall patterns. Second, **machinery parts and process infrastructure**—including open lubricant trays, condensate collection pans, drainage channels associated with cooling towers, and hydraulic system overflow reservoirs—generate inadvertent aquatic microhabitats that are rarely recognized as *Ae. aegypti* infestation and dengue risk factors during routine equipment maintenance. Third, **landscaping and green-space elements** such as ornamental ponds, drip irrigation emitters, plant saucers, and drainage sumps in factory gardens or perimeter vegetation constitute significant outdoor resting and breeding sources, particularly during periods of heavy rainfall or irrigation mismanagement. Fourth, **recyclable and waste materials**—including discarded plastic containers, packaging materials, vehicle tires used as perimeter markers, and construction debris—represent perhaps the most overlooked category of larval habitat in industrial solid waste management systems [22, 50].

### 4.3. *Aedes* Infestation and Dengue Risk Perception Among Industrial Workers

*Ae. aegypti* is highly adaptable at exploiting industrial indoors and premises, rendering any susceptible industrial workers exposed to its bites in the absence of protective behaviors or preventive measures. Receptive workplace settings—such as factory floors, warehouses, packaging halls, and canteen facilities with poor ventilation and numerous water utilities—can sustain indoor resting of *Ae. aegypti* populations that blood‐feed on stationary workers throughout working hours, creating occupational transmission pathways besides residential exposure. Thus, the dengue risk perception is a prerequisite for those susceptible workers to safeguard themselves. Risk perception—defined as the subjective assessment of the probability (perceived susceptibility) and severity (perceived seriousness) of an adverse health event—is a central determinant of health protective behavior [51]. If any industrial workers possess perceived susceptibility (belief that one can contract dengue) and perceived severity (belief that dengue represents a serious health threat), they are believed to engage in perceived benefits of preventive action, perceived barriers to action, and self‐efficacy with vector control behaviors that can reduce, prevent, or minimize health threats [16, 18, 52].

In addition, barriers to dengue prevention behavior in industrial settings extend beyond individual risk perception to encompass structural and organizational determinants. These include inadequate time allocation for vector control activities within production schedules, unclear delineation of responsibilities between facility management, occupational health officers, insufficient training of factory safety personnel in dengue risk assessment, and the absence of dengue‐specific performance indicators within existing safety management systems. Compared with other workplace hazards such as chemical exposure or machinery accidents, low dengue risk perception is often related to the relatively low case fatality rate of dengue, leading to systematic underestimation of dengue’s contribution to absenteeism, reduced productivity, and healthcare costs.

### 4.4. Factory‐Level LSM

The effectiveness of LSM in industrial settings depends critically on workforce capacity and inter‐departmental coordination. Community‐based dengue control programs highlight that the sustainability of container inspection and source elimination activities is contingent on community ownership, regular training, and feedback on monitoring results. Factory‐level LSM programs require designated trained personnel—potentially SHE teams, occupational health officers, or dedicated dengue vector control focal points—empowered with clear mandates and authorities and defined *Aedes*‐breeding inspection schedules that are integrated into routine inspection and sanitation monitoring. More significantly, dengue risk assessment based on routine entomological surveillance data can help escalate preparedness and response to preventing or interrupting dengue transmission foci in industrial areas.

In practice, factory‐level LSM programs require SHE officers to ensure compliance with environmental regulations regarding water management, waste disposal, and integrated vector management, in coordination with other management and technical experts as key personnel involved in LSM. Municipal vector control focal points/coordinators (or dedicated public health officers at local authorities) oversee the entire factory‐level LSM program implementation and reporting that comply with a local legal framework as described later. Entomologists (at regional authorities) utilize baseline dengue vector surveillance data to analyze entomological indices that determine the level of *Aedes* infestation associated with dengue risk and the level of possible insecticide resistance in *Aedes* vector populations, map breeding sites, and monitor the efficacy of larvicides. Public health professionals/epidemiologists (at provincial and regional authorities) assess health risks, track dengue transmission foci in industrial areas, and evaluate the effectiveness of dengue vector control measures and surveillance activities. This human resource and governance architecture is an integral component of the proposed D‐FAF as described later in detail.

## 5. Regulation and Standards Landscape for Workplace Dengue Prevention

The regulatory environment governing occupational health and safety in dengue‐endemic countries provides a foundational, if currently underutilized, framework for institutionalizing dengue prevention in industrial settings. The International Labour Organization (ILO) Convention No. 155 on Occupational Safety and Health (OSH) [19] and its accompanying Recommendation No. 164 establish the principle that employers bear a primary duty to assess and mitigate all risks to workers’ health arising from the work environment, including biological hazards [19]. Although most national OSH regulations derived from these instruments focus predominantly on chemical, physical, and ergonomic hazards, their scope is generally broad enough to encompass vector‐borne biological risks when interpreted in conjunction with a comprehensive legal framework of Acts, Ministerial Regulations, and Notifications, that is, as designed to empower public health officials to respond to outbreaks and manage quarantine measures.

In Thailand, the Occupational Safety, Health, and Environment Act B.E. 2554 (2011) is the cornerstone of a legal framework designed to mandate employers to control occupational health hazards and maintain safe working environments, implementing ministerial regulations specifying risk assessment, hazard communication, and corrective action requirements. A legal framework for dengue vector control by local governmental agencies [53], in conjunction with the Public Health Act, B.E. 2535 (1992), provides “additional normative guidance.” These instruments, which refer to supplementary rules, principles, standards, or expectations, are provided to direct behaviors of a building owner or even a building manager, decision‐making, or performance toward a desired, prescribed outcome to reduce or eliminate *Aedes* larval sources in buildings and surroundings [53]. However, all these instruments lack the specificity required for systematic factory‐level implementation—particularly with respect to dengue vector surveillance and control in industrial settings, for example, entomological surveillance thresholds, container inspection protocols, and performance‐based scoring criteria. Similarly, in Malaysia, the Occupational Safety and Health Act 1994 and the Environmental Quality Act 1974 collectively address workplace hygiene and waste management, but no gazette instrument specifically operationalizes dengue vector control as a mandatory employer obligation in high‐risk areas [54].

**FIGURE 3:**
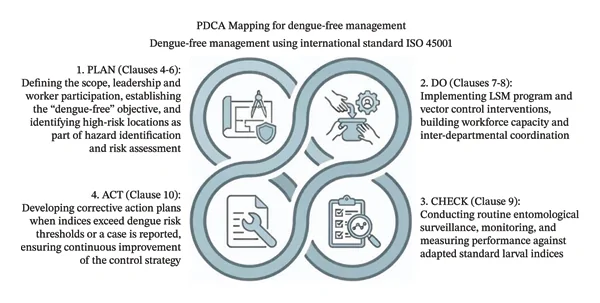
PDCA mapping for dengue‐free management in industrial sectors. By using the ISO 45001 structure, it systematically manages biological risks through the PDCA cycle for sustainability.

Voluntary management system standards offer an alternative and potentially more tractable pathway to institutionalization. ISO 45001:2018—the international standard for occupational health and safety management systems—provides a process‐based framework requiring organizations to identify hazards, assess risks, implement controls, and monitor performance through a continuous improvement cycle [21, 55, 56]. The “Plan‐Do‐Check‐Act” (PDCA) logic of ISO 45001 is directly compatible with a proposed, structured dengue assessment framework. PDCA mapping for dengue‐free management aligns with ISO 45001 clauses (Figure 3). **“PLAN (Cluses 4-6)”**—Defining the scope, leadership, and worker participation, establishing the “dengue‐free” objective, and identifying high‐risk locations (e.g., stagnant water reservoirs or potential breeding containers) as part of hazard identification and risk assessment. “**DO (Clauses 7-8)”**—Implementing LSM program and vector control interventions, building workforce capacity and inter‐departmental coordination. **“CHECK (Clause 9)”**—Conducting routine entomological surveillance, monitoring, and measuring performance against adapted standard larval indices. **“ACT (Clause 10)”**—Developing corrective action plans when indices exceed dengue risk thresholds or a case is reported, ensuring continuous improvement of the control strategy. Integration of dengue‐free criteria into ISO 45001 certification audits or supply chain social compliance audits could substantially elevate the profile and accountability of workplace vector control programs [20, 53, 54]. This approach creates accountability that usually does not exist in corporate social responsibility (CSR) or environmental, social, and governance (ESG) reports alone [55, 56], especially if third‐party auditors are looking for breeding sites as part of a compliance check.

## 6. Conceptual Foundations of the D‐FAF

### 6.1. Framework Rationale and Design Principles

In the context of the regulatory and standards landscape for workplace dengue prevention as mentioned above, all stakeholders involved in factory‐level LSM program implementation ought to understand what the concept of the D‐FAF is and its operationalization and to cooperate with SHE and OSH management systems [55, 56]. The D‐FAF is conceived as a proactive, structured, and performance‐based tool that translates evidence from dengue epidemiology, vector ecology, behavioral science, and SHE management into a practical assessment instrument applicable to diverse industrial settings in geographically different industrial areas. Currently, many industries align with ISO 45001 (Health & Safety) and ISO 14001 (Environmental) standards. Based on ISO 45001/14001 standards, a successful D‐FAF can benefit its operations on a robust SHE management system through its seven core elements: (a) leadership and commitment; (b) hazard identification and risk assessment (HIRA); (c) planning and goal setting; (d) organizational structure and competence; (e) operational controls; (f) monitoring and measurement; and (g) management review, by which it operates on the PDCA model.

The D‐FAF is grounded in three overarching design principles. First, it is proactive rather than reactive (responding to dengue cases among workers after the incident). D‐FAF aims to establish preventive receptive environmental conditions and benchmarks that prevent the risk for dengue transmission before outbreaks occur in an industrial area. Second, it is system‐integrated. D‐FAF is designed for alignment with an existing robust SHE management system, particularly ISO 45001, enabling seamless incorporation into certification processes and audit protocols. Third, it is scalable and context‐sensitive (while articulating universal principles and minimum standards). D‐FAF accommodates variation in factory type, size, and geographic setting of factories in Thailand according to the Factory Act, B.E. 2535 (1992), Ministerial Regulations, and their amendments (Supporting File A), as well as in compliance with other legal frameworks in developing countries. More interestingly, it operates on resource availability through tiered compliance levels and contextually adapted assessment criteria.

Moreover, this scalability principle is particularly important for small and medium‐sized enterprises (SMEs) outside industrial zones/estates/parks, which constitute a large share of industrial establishments in dengue‐endemic economies yet frequently lack the technical expertise and financial resources required to conduct routine entomological surveillance or to operate comprehensive occupational health programs. To avoid becoming a compliance burden that SMEs cannot realistically meet, D‐FAF is designed with a relaxed tiered‐compliance entry level (e.g., a simplified Bronze pathway) that emphasizes low‐cost source reduction and basic awareness rather than full in‐house entomological capacity. However, a Dengue‐Free Factory Score with minimum entomological thresholds derived from the acceptable larval indices adapted for SMEs needs to be developed (Supporting File C). Resource–sharing arrangements can further lower the barrier to participation: pooled or shared vector–control services organized at the level of the industrial estate, technical support from local authorities and the regional vector–control authority (including periodic entomological audits by trained officers), and low‐cost digital tools such as mobile‐based container‐inspection reporting and shared larval‐index dashboards (Section 7) can collectively substitute for capabilities that individual SMEs cannot maintain alone. In this way, the framework articulates universal minimum standards while remaining attainable across the full range of enterprise sizes.

Applicability of D‐FAF is not limited to Thailand. While the framework was developed drawing primarily on Thailand’s regulatory and epidemiological context, several of its core components are expected to be broadly applicable across dengue‐endemic countries: the five assessment domains (Section 6.2), the PDCA‐based continuous improvement logic aligned with ISO 45001, and the underlying principle that structured workplace‐level LSM should be integrated into SHE systems. By contrast, other elements are likely to require local adaptation, namely (i) national occupational health and safety legislation and certification pathways, which vary in scope and enforceability across jurisdictions; (ii) industrial infrastructure and factory typologies, which differ in scale, water‐storage practices, and production processes across regions; and (iii) local vector ecology, including species composition (e.g., relative contributions of *Ae. aegypti* vs. *Ae. albopictus*), insecticide resistance profiles, and container preferences, which together shape which entomological indices and thresholds are epidemiologically meaningful. Countries seeking to adopt D‐FAF are therefore encouraged to retain its core domain structure and PDCA logic while recalibrating specific indicators, weights, and certification thresholds (Table 1, Table 2) to reflect national regulatory frameworks and local entomological evidence.

**TABLE 1:** Proposed (tentative) weighting scheme^a^ and preliminary scoring rubric for the five D‐FAF assessment domains.

Assessment domain	Weight (%)	Example sub‐indicators	Scoring method
Environmental and Container Risk Assessment	30	Industrial‐adapted BI/CI/HI; presence of larvae/pupae in large‐volume utilities; container audit coverage	Index‐based bands were converted to a 0–100 sub‐score, then weighted
Structural and Engineering Controls	20	Tank/cistern screening; drainage design and maintenance; solid‐waste management; landscaping practices	Checklist compliance (% of controls in place)
Surveillance and Response Capacity	20	Syndromic surveillance; case reporting protocols; worker notification and treatment access	Capability rubric (absent/partial/full)
Organizational Governance and Policy	15	Written dengue policy; designated trained personnel; integration into SHE plan; inter‐departmental coordination	Documentary evidence checklist
Workforce Knowledge, Attitudes, and Practices (KAP)	15	Perceived susceptibility/severity; benefits/barriers; self‐efficacy (Health Belief Model)	Validated KAP questionnaire (mean % score)
Total	100	Composite Dengue‐Free Factory Score	Weighted sum ⟶ tier (Table 2)

^a^Weights are provisional and require psychometric testing and external validation before adoption. These values are proposed by the opinion of experts and authors, not yet evidence‐based by validation studies.

**TABLE 2:** Proposed (tentative) quantitative criteria^a^ for the three D‐FAF certification levels, combining the composite dengue‐free factory score with minimum entomological thresholds.

Certification level	Composite dengue‐free factory score (%)	Minimum entomological criteria (industrial‐adapted)	Illustrative interpretation
Bronze (baseline)	60–74	BI ≤ 20; CI ≤ 10%; HI ≤ 10% (premise‐level), with no larvae in large‐volume water utilities	Functional prevention infrastructure and basic awareness in place; residual breeding tolerated but monitored
Silver (intermediate)	75–89	BI ≤ 5; CI ≤ 5%; HI ≤ 5%, sustained across at least two consecutive inspection cycles	Sustained vector control and a trained workforce; entomological risk consistently low
Gold (full)	≥ 90	BI ≤ 1; CI ≈ 0%; HI ≤ 1%, with no positive large‐volume containers and no autochthonous worker case in the preceding cycle	Minimal entomological risk, comprehensive governance, and exemplary surveillance and response capacity

^a^All values are preliminary and require field validation when applied to industrial zones/estates/parks. These values are proposed by the opinion of experts and authors, not yet evidence‐based by validation studies.

### 6.2. Core Domains of Assessment

**FIGURE 4:**
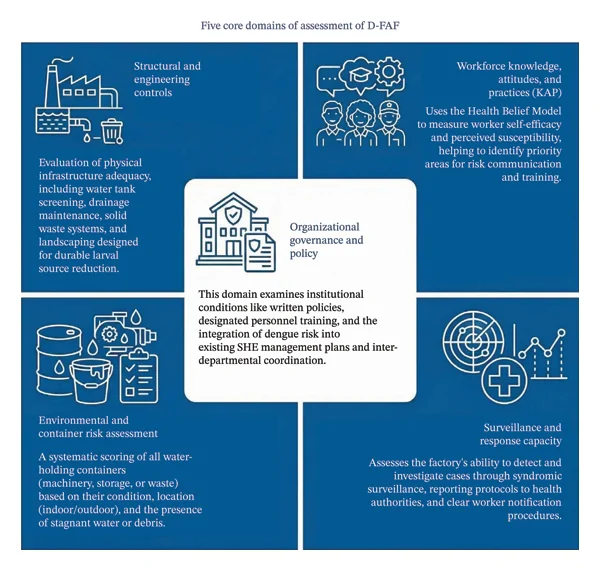
D‐FAF: a comprehensive framework for dengue prevention in industrial sectors.

D‐FAF is organized around five core assessment domains reflecting the principal determinants of dengue transmission risk in industrial settings (Figure 4). The first domain, **Organizational Governance and Policy**, examines the institutional conditions enabling sustainable dengue prevention, including the existence of written dengue prevention policies, designation and training of responsible personnel, integration of dengue risk into SHE management plans, inter‐departmental coordination mechanisms, and engagement with local authorities and other health authorities. This domain reflects recognition that individual behavioral change is insufficient without supportive organizational structures and accountability mechanisms [20, 21, 54–56].

The second domain, **Structural and Engineering Controls**, evaluates the physical adequacy of dengue‐prevention infrastructure, including screening of water tanks and storage vessels, drainage system design and maintenance, solid waste management systems, and landscaping practices. This domain draws on principles of environmental design for vector control, recognizing that structural modifications often provide more durable and cost‐effective larval source reduction than behavioral interventions alone.

The third domain, **Environmental and Container Risk Assessment**, encompasses systematic inspection and scoring of all water‐holding containers within industrial premises—classified by type (water storage, machinery, landscaping, and recyclable waste), condition (presence of larvae or pupae, stagnant water, and organic debris), and location (indoor, outdoor, and perimeter zones). Factory‐adapted entomological indices (Supporting File C) provide quantitative risk benchmarks analogous to WHO‐recommended surveillance thresholds for residential settings [44].

It should be emphasized that the conventional larval indices on which this domain relies—the BI, CI, and HI—were originally developed for residential settings and therefore have important limitations when applied to large industrial settings inside industrial areas versus individual factories outside industrial areas (Supporting File C). The “house” or premise denominator that underlies BI and HI does not map cleanly onto a single factory footprint, which may span extensive grounds with a disproportionately dense and heterogeneous inventory of containers; expressing larval positivity per “premise” can thus either understate or overstate risk depending on how the unit is defined. In addition, standard indices treat each positive container equally and do not weight by productivity, so they fail to capture the outsized epidemiological contribution of large‐volume habitats such as cooling sumps and fire‐suppression cisterns that can sustain very high pupal output. To address these limitations, factory‐adapted denominators—for example, indices expressed per unit floor or ground area, per number of workers, or per production unit—and pupal‐ or productivity‐weighted measures should be considered as supplementary metrics alongside the conventional indices (Supporting File C). Because no industrial‐specific index has yet been validated, the development and field validation of factory‐specific entomological indices is identified as a key research gap (Section 7), and the thresholds used in this framework should be regarded as provisional pending such validation.

The fourth domain, **Workforce Knowledge, Attitudes, and Practices (KAP)**, operationalizes the Health Belief Model as an assessment lens for gauging individual and collective readiness to engage in dengue prevention behaviors. Assessment instruments adapted from validated KAP tools measure workers’ perceived susceptibility, perceived severity, perceived benefits and barriers, and self‐efficacy with respect to dengue prevention activities. KAP scores are used both diagnostically—to identify priority areas for risk communication and training—and as performance indicators tracked over time.

The fifth domain, **Surveillance and Response Capacity**, assesses the factory’s capability to detect, investigate, and respond to dengue cases among workers in a timely manner. This includes syndromic surveillance systems, reporting protocols to occupational health personnel SHE teams, public health authorities, and procedures for worker notification and treatment access. Robust response capacity is essential to prevent workplace dengue clusters from amplifying into broader community transmission events in industrial areas.

### 6.3. Scoring, Certification, and Continuous Improvement

This proposed D‐FAF employs a weighted scoring system across its five domains, reflecting the relative epidemiological importance of each in driving dengue transmission risk in industrial settings inside industrial areas rather than individual factories outside industrial areas. Such proposed domain scores are aggregated into a composite Dengue‐Free Factory Score, which maps to three certification levels: Bronze (baseline compliance, indicative of functional dengue prevention infrastructure and awareness), Silver (intermediate compliance, demonstrating sustained vector control and trained workforce capacity), and Gold (full compliance, reflecting minimal entomological risk, comprehensive governance, and exemplary surveillance and response capacity). This tiered structure under review is intended to recognize and reward incremental progress, maintaining engagement even among factories facing resource constraints, while creating aspirational benchmarks for industry leaders. To operationalize these tiers, we propose tentative quantitative criteria that combine an aggregate Dengue‐Free Factory Score with minimum entomological thresholds derived from the standard larval indices adapted for industrial settings (BI, CI, and HI). The aggregate score is computed as the weighted sum of the five assessment domains (Section 6.2), with the Environmental and Container Risk Assessment domain assigned the greatest weight given its direct role in driving transmission risk, followed by Structural and Engineering Controls and Surveillance and Response Capacity, then Organizational Governance and Policy and Workforce KAP. The proposed domain weights and their rationale are presented in Table 1, and the resulting score bands with accompanying entomological ceilings for each certification level are summarized in Table 2.

The weighting rationale prioritizes domains most proximal to transmission risk: direct entomological evidence (Environmental and Container Risk Assessment) carries the highest weight, followed by the engineering controls and surveillance capacity that determine whether breeding is prevented and cases are detected, with governance and workforce KAP serving as enabling conditions. This rubric is proposed to support external validation and has not yet undergone psychometric testing; the weights, sub‐indicators, and scoring methods should be empirically calibrated and validated before the rubric is used for certification, consistent with the research gap on KAP‐instrument validation noted in Section 7.

As an illustrative example, consider an industrial estate or zone that scores 82% overall—strong on structural controls and surveillance but weaker on workforce KAP—and records BI = 4, CI = 4%, and HI = 3% at audit, with *Ae. aegypti* larvae detected in its cooling sumps or fire cisterns. This facility would meet the Silver threshold but fall short of Gold because its entomological indices and KAP domain remain above the Gold ceilings; targeted risk communication and elimination of the residual small‐container breeding would be the corrective actions needed to progress. The score bands and entomological ceilings presented here are deliberately proposed as tentative starting values; the relative domain weights, the precise cutoffs, and the industrial‐adapted index thresholds all require empirical calibration and field validation across diverse factory types before adoption as a certification standard. Taken together, it is important that the weighting scheme and certification thresholds for the D‐FAF framework were preliminary and intended for conceptual demonstration only unless they are supported by further published validation studies.

The PDCA cycle underpinning ISO 45001 provides the continuous improvement logic for D‐FAF implementation. Annual re‐assessment against D‐FAF criteria—ideally conducted by trained external auditors or national/regional/provincial vector control authority officers—generates actionable corrective action plans that feed back into facility management plans. Aggregate factory‐level data from D‐FAF assessments across a regional industrial zone can further serve as an epidemiological early warning system, identifying geographic clusters of high entomological risk before clinical dengue cases emerge.

### 6.4. Alignment With National and International Standards

The D‐FAF is explicitly designed for alignment with multiple normative frameworks. Its environmental management domain aligns with ISO 14001:2015 criteria for identifying and controlling environmental hazards. Its workforce training and communication components are consistent with ILO OSH Convention 155 and Recommendation 164 requirements for worker information and education on occupational risks. Its governance domain incorporates elements from the WHO’s Global Vector Control Response (GVCR) 2017–2030 [57], particularly the call for multisectoral engagement and capacity building beyond traditional health sector boundaries. By articulating these cross‐framework compatibilities, D‐FAF positions dengue prevention not as an isolated public health intervention but as an integral element of responsible corporate ESG performance—an increasingly important dimension of investor and supply chain stakeholder scrutiny.

It is important to clarify that D‐FAF is conceived as a complement to, rather than a substitute for, the WHO GVCR 2017–2030 and broader IVM strategies [57]. IVM provides an overarching, cross‐sectoral strategic approach to vector control at the national and community levels, encompassing multiple vector species, transmission settings, and control methods coordinated across health, environment, and other sectors. D‐FAF, by contrast, operationalizes IVM principles specifically at the level of the individual industrial premise, translating the broader IVM emphasis on evidence‐based decision‐making, multisectoral collaboration, and integrated methods into a standardized, auditable, performance‐based assessment instrument embedded within existing SHE management systems. In this sense, D‐FAF can be understood as a workplace‐level implementation mechanism that extends the reach of national IVM programs into industrial settings that are not always well served by community‐based vector control activities while remaining fully consistent with and reinforcing IVM’s core principles.

## 7. Research Gaps and Future Directions

In 2026, the Department of Disease Control (DDC), Ministry of Public Health, initiated the deployment of D‐FAF at different levels of health sectors. Meanwhile, cooperating with the D‐FAF aligned with a robust SHE management system in industrial settings across the country requires several important evidence gaps that constrain the immediate operationalization of D‐FAF and define a research agenda for the field.

First, there is a paucity of published epidemiological data quantifying the proportion of dengue cases attributable to occupational exposure in industrial settings, as most surveillance systems do not distinguish workplace from residential transmission. Prospective occupational cohort studies with entomological co‐surveys at enrolled factories are needed to establish transmission attribution baselines and validate the epidemiological relevance of factory‐specific larval indices. Second, the feasibility, acceptability, and cost‐effectiveness of D‐FAF implementation across diverse factory types—including small and medium enterprises with limited SHE resources—require prospective evaluation through pilot studies in multicountry settings. Third, the behavioral science foundations of D‐FAF require empirical validation of KAP instruments specifically calibrated for industrial worker populations across different cultural and linguistic contexts. Their psychometric properties may differ substantially in occupational populations characterized by varying education levels, employment precarity, migrant status, and organizational culture. Fourth, the role of digital technologies—including mobile‐based container inspection reporting, GPS‐referenced larval index mapping, and data dashboard interfaces for factory management—in enhancing the efficiency and sustainability of D‐FAF implementation warrants systematic investigation. Digital entomological surveillance platforms have shown promise in community settings and could be adapted and validated for factory‐level deployment.

Finally, within the public health context and laws, the governance and regulatory pathways for formalizing D‐FAF as a national standard or certification scheme require multistakeholder engagement among the DDC, Ministries, the Industry Estate Authority of Thailand, industry associations, local authorities, provincial public health authorities, occupational safety regulators, and international standards bodies. Cross‐sectoral policy dialog processes—informed by evidence from pilot implementation—will be essential to translate the D‐FAF concept into institutionalized practice at national and regional scales.

To strengthen the D‐FAF framework and provide its clear research pathway, the general proposal for external validation can be operationalized through a structured, three‐phase methodology. This involves multisectoral pilot testing, statistical calibration, and rigorous benchmarking against established epidemiological indicators.

### 7.1. Phase 1: Multisectoral Pilot Implementation

Validation must span diverse manufacturing environments to ensure the framework adapts to different operational realities, spatial layouts, and worker demographics.

i. **Sector selection:** Run parallel pilots in high‐risk industrial sectors with distinct structural characteristics (e.g., electronics, textile manufacturing, heavy machinery, and chemical processing).
ii. **Geographic diversity:** Deploy the rubric across varied industrial zones/estates/parks, balancing high‐density urban industrial areas with expansive suburban or rural areas.
iii. **Operational scale:** Include both multinational corporations (typically high‐resource) and SMEs to test rubric viability under resource constraints.

### 7.2. Phase 2: Benchmarking Against Epidemiological Indicators

To prove its real‐world utility, the composite Dengue‐Free Factory Score must demonstrate a statistically significant correlation with localized dengue transmission data.

i. **Retrospective comparison:** Match historical dengue case counts and localized cluster data from local health registries with the industrial areas’ initial baseline scores.
ii. **Prospective cohort study:** Track the incidence of confirmed dengue cases among the workforce over a 12‐to‐24‐month period following D‐FAF implementation.
iii. **Vector density mapping:** Correlate higher D‐FAF certification tiers (Silver/Gold) with a measurable, sustained drop in traditional local vector surveillance metrics, such as the Ovitrap Index (OI) and Adult Productivity Assays (Supporting File C).

### 7.3. Phase 3: Statistical Calibration and Construct Validation

Before the rubric is locked as a certification standard, the internal mechanics of the scoring system require empirical tightening.

i. **Psychometric testing for KAP:** Run exploratory and confirmatory factor analyses (EFA/CFA) on the workforce KAP survey instruments to ensure questions reliably measure intended behaviors.
ii. **Sensitivity analysis:** Model how shifts in individual domain weights (e.g., increasing the weight of structural controls over governance) impact overall certification distributions.
iii. **Receiver operating characteristic (ROC) curves:** Use ROC analysis to determine the exact aggregate score cutoffs and entomological ceilings that optimize the framework’s sensitivity and specificity in predicting a “dengue‐safe” environment.

## 8. Conclusion

The convergence of dengue persistence and industrial expansion, if accompanied by rapid, uncontrolled, unplanned urbanization, creates an urgent and underappreciated occupational health challenge across endemic countries, including Thailand. Factories and manufacturing premises, with their diverse inventory of unmanaged water‐holding containers and dense, highly mobile workforces, represent significant yet systematically neglected contributors to local dengue transmission. The D‐FAF proposed in this review offers a conceptually grounded, evidence‐informed, and operationally feasible approach to addressing this gap. By integrating entomological surveillance, structural engineering controls, workforce behavioral interventions, organizational governance measures, and active case surveillance into a unified performance‐based system—and by explicitly aligning with established SHE management standards—D‐FAF creates the conditions for sustained, scalable, and accountable dengue prevention in the industrial workplace. Implementation and validation of D‐FAF across diverse factory settings in dengue‐endemic countries will be a critical next step toward protecting worker health, reducing business disruption, and contributing meaningfully to national and subnational dengue control targets.

## Funding

No funding was received for this manuscript.

## Conflicts of Interest

The authors declare no conflicts of interest.

## Supporting Information

Additional supporting information can be found online in the Supporting Information section.

## Supporting information


**Supporting Information 1** Supporting File A: Industrial area.

**Supporting Information 2** Supporting File B: Review methods and synthesis for narrative review.

**Supporting Information 3** Supporting File C: Entomological surveillance methods for individual factories outside industrial area.

**Supporting Information 4** Supporting Table 1 Factors driving the paradox.

## Data Availability

The data that support the findings of this study are available on request from the corresponding author. The data are not publicly available due to privacy or ethical restrictions.
