# Supplementary material for: Proactive Initiative to Establish a Dengue‐Free Factory Assessment Framework in Dengue High‐Risk Industrial Areas: A Narrative Review
Source: Interdiscip Perspect Infect Dis. 2026 Sep 6;2026:4901627. doi: 10.1155/ipid/4901627 (PMC13547597; doi:10.1155/ipid/4901627)
Supplement: Supplementary file 1 [file IPID-2026-4901627-s001.pdf]

Review Methods and Synthesis for Narrative Review

General Consideration of Review Methods

This article published by Ritthison et al (2026) is a narrative review. Although it does not follow a systematic-review protocol, the supporting literature was identified through a structured search to ensure transparency and reproducibility.

Database searched

We searched PubMed, Scopus, and Web of Science for peer-reviewed publications, supplemented by targeted retrieval of authoritative grey literature from the World Health Organization (WHO), the International Labour Organization (ILO), and the International Organization for Standardization (ISO).

Search Terms and Iterative Process

The principal search terms or keywords were combined using Boolean operators and included "dengue," "*Aedes aegypti*," "larval source management," "dengue vector control," "entomological indices," "vector surveillance," "occupational health," "workplace," "industrial area," "industrial setting," "factory," "ISO 45001," "ISO 14001," and "safety, health and environment," together with their common synonyms.

Boolean operators are connecting words (AND, OR, NOT) used in search engines and databases to define relationships between keywords. These three core operations were always typed in ALL CAPS, ensuring the database recognizes them as logic commands rather than regular keywords. They are essential for narrowing or broadening literature search to find highly relevant information or fine-tune results.

Iterative process that operated through a custom Boolean search string started with a broad search using OR to group synonyms and related concepts together, then sequentially add main keywords or specified terms with AND to combine different concepts, or with NOT to filter out irrelevant topics or specific sub-disciplines.

Inclusion and Exclusion Criteria

Sources were included if they addressed dengue epidemiology, vector ecology and larval source management, dengue risk in occupational or industrial settings, workers' risk perception, or the regulatory and management-system landscape relevant to workplace vector control.

Priority was given to original research, systematic reviews, and normative or standards documents published in English.

Studies were excluded if they were not retrievable in full text, were unrelated to dengue or its vectors, or did not inform any of the framework's evidence domains.

Emphasis was placed on literature published from 1980 onward to reflect contemporary epidemiological and vector-control understanding, while seminal earlier works (e.g., foundational descriptions of entomological indices and the Health Belief Model) were retained where they remained conceptually relevant.

Synthesis

Unlike a systematic review, a narrative review synthesized diverse concepts, theories, and contexts by identifying overarching patterns and critical gaps.

Therefore, synthesis in a narrative review that answers the objectives did not structure by summarizing individual studies. Instead, the authors group fine-tuned literature by building themes or concepts to weave fine-tuned search results into the same sub-topics or sub-themes. This ensures chronological evolution of themes or concepts (e.g., showing how a theory or phenomenon changed over time), and methodological approaches (e.g., comparing qualitative findings against quantitative results).

The evidence drawn from this search underpins the framework synthesized in the following sections and corresponds to references [1]–[57].

Citation:

Ritthison W, Bhumiratana A, Padungtod C, Tongtummachart N. Proactive Initiative to Establish a Dengue-Free Factory Assessment Framework in Dengue High-Risk Industrial Areas: A Narrative Review. Interdisciplinary Perspectives on Infectious Diseases, 2026;2026:4901627.
