# Supplementary material for: Proactive Initiative to Establish a Dengue‐Free Factory Assessment Framework in Dengue High‐Risk Industrial Areas: A Narrative Review
Source: Interdiscip Perspect Infect Dis. 2026 Sep 6;2026:4901627. doi: 10.1155/ipid/4901627 (PMC13547597; doi:10.1155/ipid/4901627)
Supplement: Supplementary file 2 [file IPID-2026-4901627-s002.pdf]

Supplementary Table 1 Factors driving the paradox

**Factors**	**Impact on public health and epidemiology**
**Complex viral dynamics and immunological factors**	In hyperendemic regions, the simultaneous co-circulation of all four DENV serotypes (DENV-1 to DENV-4) sustains cycles of infection and transmission. This leads to a cyclic pattern of outbreaks where different serotypes take turns predominating, and wanning cross-immunity, as described in detail in the text. Once a susceptible person becomes infected with one DENV serotype, DENV provides only partial, temporary immunity to the others. Subsequent infection with a different serotype increases the risk of severe dengue (DHF/DSS).
**Lack of specific treatment and vaccine limitations**	The lack of specific anti-viral treatments and significant limitations in currently available vaccines are major barriers to controlling dengue, allowing DENV to persist within endemic countries despite extensive dengue control efforts. Thus, the current surge as of 2025–2026 can jeopardize the management of dengue as it remains largely reactive rather than proactive. Case management relies on supportive care and early detection to reduce fatality rates. Meanwhile, the application of dengue vaccines is complicated by the risk of antibody-dependent enhancement, where a second infection with a different serotype can lead to more severe disease. This process leads to heavy viremia and can complicate the host's ability to clear the new infection effectively.
**Environmental and climate drivers**	Climate variability directly influences dengue transmission. Rising temperatures, high humidity, and increased precipitation from climate change (such as El Niño events) create ideal conditions for the upregulation of the proliferation of *Ae. aegypti* vector population and the spread of DENV. The physical environment of modern cities acts as a force multiplier for these climatic drivers. As a result, these environmental and climate drivers dictate the survival, reproduction, and geographic range of the *Aedes* vectors (*Ae. aegypti* and *Ae. albopictus*) as creating perennial reservoirs of infection.
**Vector adaptability and resistance**	The adaptability and resistance of *Aedes* vectors are primary drivers of dengue persistence, as they allow vector populations to survive control efforts and thrive in new environments. Particularly, *Ae. aegypti* is adapted well to urban environments and the propensity of developed resistance to insecticides commonly used in vector control as described in detail in the text.
**Rapid urbanization and infrastructure gaps**	Unplanned urban expansion leads to high density of susceptible human hosts and human-dominated environments favoring year-round breeding sites for *Ae. aegypti*. When urbans or industrial areas grow faster than their infrastructure can create the resulting gaps—specifically in low-quality housing, inadequate sanitation, poor waste

	disposal, and poor water storage practices—and then become the primary drivers of perennial reservoirs of the infection.
**Migration and human mobility**	While *Aedes* vectors transmit DENV locally, human mobility is the primary driver of its long-distance dispersal and regional persistence. Because humans are the main reservoir for the virus, their movement—ranging from daily commutes to international travel—seeds new outbreaks and maintains viral circulation in areas where existing low or very low prevalence or low level of natural immunity within the population and otherwise disappearance of DENV.
