# Supplementary material for: Proactive Initiative to Establish a Dengue‐Free Factory Assessment Framework in Dengue High‐Risk Industrial Areas: A Narrative Review
Source: Interdiscip Perspect Infect Dis. 2026 Sep 6;2026:4901627. doi: 10.1155/ipid/4901627 (PMC13547597; doi:10.1155/ipid/4901627)
Supplement: Supplementary file 3 [file IPID-2026-4901627-s003.pdf]

Entomological Surveillance Methods for Individual Factories Outside Industrial Area

As mentioned by the Ritthison et al (2026), conventional larval indices like the House Index (HI) and Breteau Index (BI) are designed for residential areas. They fail at an individual factory outside an industrial area (whether industrial zone, industrial estate, or industrial park) due to large footprints, sparse human presence, and disconnected spatial data, but work inside an industrial estate due to dense, controlled, and uniform infrastructure.

Why They Fail at an Individual Factory Outside an Industrial Area

- **Math Denominator Mismatch:** The core calculation of HI and BI requires "number of houses inspected". Factories do not conform to residential housing definitions, making the denominator arbitrary and skewing the resulting index compared to surrounding community transmission data.
- **Host Dispersion:** Vectors (like *Aedes aegypti* mosquitoes) rely on dense human populations to bite and reproduce. Individual factories isolated on large lots have vast spaces between buildings with few workers, meaning mosquito populations behave differently than in clustered, multi-building residential zones.
- **Cryptic and Spatially Fragmented Breeding Sites:** Factories present vast peridomestic settings (e.g., cooling towers, massive drainage networks, stormwater drains) that are vastly different from residential water-holding containers. Traditional larval indices fail to capture this vastly different spatial and container ecology.

Why They Work Well for Factories Confined Within an Industrial Area

- **Homogeneous Facilities:** Factories within an industrial estate are built as dense, uniform units in close proximity to one another. They share very similar water management and environmental infrastructure, allowing inspectors to treat factory buildings like the "residential blocks" the indices were originally calibrated for.
- **Clustered Human Density:** Because an industrial estate groups many facilities and their employees together, the population density mimics urban residential zones. The mosquito biting and resting patterns become stable and mathematically measurable.
- **Enclosed & Standardized Environments:** Industrial estates feature uniform, controlled perimeters, standardized water systems, and unified waste management. This allows vector control teams to accurately inspect the premises and reliably calculate positive containers per 100 "inspected units" (BI).

However, alternative entomological surveillance methods are better suited for individual factories because they measure actual vector productivity and presence without relying on residential "house" definitions.

1. Ovitraps (Egg Traps)

- **Standardized Surveillance**: Ovitraps use uniform containers to attract pregnant female mosquitoes, eliminating the bias of factory-specific structural differences.

- **Early Detection**: They detect vector presence in low-density settings long before larvae become visible in massive factory infrastructure.
- **Ovitrap Index (OI)**: Calculated as the percentage of positive traps out of the total traps deployed: OI (%) = (Positive Traps / Total Traps) x 100

2. Pupal Productivity Indices

- **Adult Production Proxy**: Counting pupae is a direct proxy for adult mosquito production because pupal mortality is exceptionally low.
- **Targeted Intervention**: Identifies the specific, massive containers (like cooling towers or stormwater drains) driving the majority of the risk.
- **Pupae per Person (PPI)**: Calculated by dividing the total number of pupae collected by the total number of workers present in that factory zone:

  PPI = Total Pupae/Total Workers

3. Adult Mosquito Traps

- **Direct Risk Assessment**: Traps like BG-Sentinel or gravid traps capture adult mosquitoes, providing immediate data on the active biting risk to workers.
- **Viral Screening**: Captured adults can be pooled and tested for viruses (like Dengue or Zika) to confirm if the factory population is a transmission hotspot.

Comparison for Individual Factory Surveillance

Method	Metric Calculated	Primary Advantage in Factories	Major Operational Challenge
**Ovitraps**	Ovitrap Index (OI)	Highly sensitive in large, low-density spaces	Requires weekly site visits to collect and count logs
**Pupal Indices**	Pupae per Person (PPI)	Pinpoints the exact infrastructure causing the risk	Labor-intensive to sample massive industrial water volumes
**Adult Traps**	Catch per Unit Effort (CPUE)	Measures actual worker exposure to biting vectors	Requires continuous power supply and specialized lures

Citation:

Ritthison W, Bhumiratana A, Padungtod C, Tongtummachart N. Proactive Initiative to Establish a Dengue-Free Factory Assessment Framework in Dengue High-Risk Industrial Areas: A Narrative Review. Interdisciplinary Perspectives on Infectious Diseases, 2026;2026:4901627.
