# Supplementary material for: Proactive Initiative to Establish a Dengue‐Free Factory Assessment Framework in Dengue High‐Risk Industrial Areas: A Narrative Review
Source: Interdiscip Perspect Infect Dis. 2026 Sep 6;2026:4901627. doi: 10.1155/ipid/4901627 (PMC13547597; doi:10.1155/ipid/4901627)
Supplement: Supplementary file 4 [file IPID-2026-4901627-s004.pdf]

Industrial Area, Industrial Zone, Industrial Estate, and Industrial Park

This article published by Ritthison et al (2026) employed a neutral term "**Industrial Area**", describing a geographic region designated and zoned primarily for factories, manufacturing, logistics, and warehousing. It is designed to keep heavy machinery, pollution, and large vehicle traffic away from residential neighborhoods.

In Thailand, **"Industrial Area"** acts as a legal and structural umbrella term, but only in specific ways upon Thai legal provisions such as the Factory Act or the Industrial Estate Authority of Thailand (IEAT) Act.

Therefore, the terms–**"Industrial zone"**, **"Industrial estate"**, and **"Industrial park"**–are often used interchangeably to describe industrial areas designated for manufacturing and logistics. They carry distinct regulatory, ownership, and scale differences.

Key Differences at a Glance

Industrial Zone:

A broad geographic region or city-wide zoning designation (often spanning multiple provinces), which is dedicated to industrial growth and development. Industrial zones are typically governed directly by Ministries of Industry to boost national or regional economies.

Industrial Zones are governed by Section 30 of the Factory Act. These are privately owned lands approved by the Ministry of Industry to act as industrial zones. While they must provide public utilities similar to an estate, they remain under standard Ministry of Industry jurisdiction rather than the IEAT.

Industrial Estate:

Highly regulated, government-supervised industrial hubs. Industrial estates are overseen by state enterprises like the Industrial Estate Authority of Thailand (IEAT). They function like self-contained industrial cities with built-in utility plants, customs offices, and exclusive foreign-ownership privileges. Industrial Estates are governed directly by the Industrial Estate Authority of Thailand Act B.E. 2522 (1979). They have their own legally autonomous, one-stop service centers. Factories inside an Industrial Estate's designated **"Industrial Area"** are automatically granted factory licenses without needing separate Ministry of Industry approval. Under the IEAT Act, **"Industrial Area"** is legally defined as a component part inside a designated Industrial Estate.

Industrial Park:

A privately developed and managed plot of land (usually smaller in scale) built specifically for manufacturing and distribution. They feature ready-to-use infrastructure but are typically overseen by private developers or the Board of Investment (BOI) rather than a state-owned authority. Industrial Parks are not explicitly defined as a separate legal tier under Thai statutory law. Legally, an **"Industrial Park"** is simply a commercial marketing term used by private

developers. To get legal and tax privileges, a private "Industrial Park" must formally register either as an **“Industrial Zone”** under the Factory Act or enter a joint-operation agreement with the IEAT to become an **“Industrial Estate”**.

Comparison of three industrial features in Thailand upon Thai legal provisions

Feature	Industrial Zone	Industrial Park	Industrial Estate
**Ownership**	Public / Government-designated	Privately developed	State-operated or joint public-private venture
**Regulation**	Governed by Ministry of Industry	Regulated by local Factory Acts / BOI	Supervised by a state authority (e.g., IEAT)
**Scope / Scale**	Broad regional or city-level areas	Smaller, dedicated sites (e.g., >500 rai or >80 hectares)	Comprehensive industrial cities with integrated facilities
**Land Ownership Rules**	Standard local zoning rules apply	Foreign land ownership usually restricted without special privileges	Foreign companies can typically buy land outright

Summary of legal power

Term	Legal Framework in Thailand	Governing Authority
**Industrial Area**	The foundational physical zone required by Thai zoning and factory laws	Ministry of Industry / IEAT
**Industrial Estate**	Regulated by the IEAT Act; highly privileged	IEAT (State Enterprise)
**Industrial Zone**	Regulated by Section 30 of the Factory Act	Department of Industrial Works, Ministry of Industry
**Industrial Park**	Marketing term; must register under the Factory Act or IEAT to have legal standing	Private Developer / Co-managed

Citation:

Ritthison W, Bhumiratana A, Padungtod C, Tongtummachart N. Proactive Initiative to Establish a Dengue-Free Factory Assessment Framework in Dengue High-Risk Industrial Areas: A Narrative Review. Interdisciplinary Perspectives on Infectious Diseases, 2026;2026:4901627.
